# Insight on the Mechanical Properties of Facile Hydrophobic-Barrier-Patterned Bacterial Nanocellulose via Self-Bonding Mechanism

**DOI:** 10.1021/acsnanoscienceau.4c00077

**Published:** 2025-03-20

**Authors:** Maurelio Cabo, Nitin More, Jeffrey R. Alston, Eric Laws, Rutujaa Kulkarni, Ram V. Mohan, Dennis R. LaJeunesse

**Affiliations:** † Department of Nanoscience, Joint School of Nanoscience and Nanoengineering, 14616 University of North Carolina Greensboro, Greensboro, North Carolina 27402, United States; ‡ Department of Nanoengineering, 3616 North Carolina Agricultural and Technical State University, Greensboro, North Carolina 27401, United States

**Keywords:** Bacterial Nanocellulose, Hot Press, Mechanical Properties, Hydrophobic Barriers, Self-Bonding Mechanism

## Abstract

Enhancing the mechanical and structural properties of bacterial nanocellulose (BNC) is key to its use in sustainable nanocomposites. This study employed a hot-press drying method with hydrophobic barriers, folding BNC into four layers and pressing with carbon fiber and Teflon sheets. At 120 °C, carbon fiber-pressed BNC achieved a tensile strength of 43.91 N/mm^2^, 13.84% higher than oven-dried samples and 43.87% higher than Teflon-pressed samples. Scanning electron microscopy (SEM), KLA-Zeta, and atomic force microscopy (AFM) analyses revealed improved self-bonding and surface roughness. Fourier transform infrared spectroscopy (FTIR) and X-ray diffraction (XRD) confirmed increased crystallinity and altered hydrogen bonding, enhancing stiffness and structural stability. Optical and thermal tests showed carbon fiber-pressed BNC was less transparent with moderate heat resistance, while Teflon-treated samples remained clear with higher thermal stability. These findings demonstrate that patterned hot pressing strengthens BNC’s self-bonding, advancing its potential for use in structural nanocomposites, flexible electronics, and biocompatible scaffolds.

## Graphical abstract

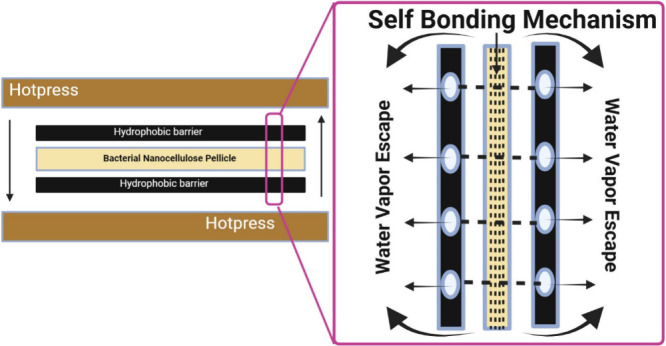

Plastic waste has become a significant environmental issue, especially in marine ecosystems, driving the demand for sustainable alternatives to petroleum-based materials.^1–3^ Among biodegradable polymers and biomass, cellulose, a highly abundant polysaccharide with an estimated annual biomass production of 1.5 × 10^12^ tons,^4^ has garnered attention due to its renewable nature and wide commercial applications. However, despite its abundance and utility, cellulose suffers from inherent brittleness and poor processability, limiting its application scope.^5,6^ To address these challenges, researchers have explored various strategies, such as functionalization^7,8^ and blending,^9,10^ to improve cellulose’s thermomechanical properties which lead to better energy absorption and resistance to deformation, which in turn increases the toughness of the material.^11,12^ However, innovative yet facile methods are still needed to enhance the toughness and versatility of cellulose-based materials.

Bacterial nanocellulose (BNC), produced by aerobic bacteria,^13^ offers significant advantages over plant-derived cellulose, including higher crystallinity,^14^ purity,^15^ and specific surface area.^16^ This biopolymer exhibits unique functional properties such as excellent mechanical strength,^17^ high optical transparency,^18^ biocompatibility,^19^ and low thermal expansion,^20^ making it a promising building block for eco-friendly materials. Unlike plant cellulose, BNC is free of hemicellulose, lignin, and pectin, which allows for its direct application in advanced materials.^21^ However, the random alignment of BNC nanofibrils during production poses challenges in fully harnessing its mechanical potential.^22^ Although manipulating intrinsic properties such as crystallinity and polymerization through culture conditions has shown promise, further innovation in processing techniques is required to optimize its properties for advanced applications. One of the facile processing strategies that researchers studied recently is to elucidate the self-bonding mechanism in various polymers.

Zhao et al. found that increasing the intrinsic molecular constant (IMC) of self-bonding natural fiber materials (SNFMs) enhances mechanical properties due to a higher presence of phenolic hydroxyl groups and an increased S–OH/G–OH ratio in lignin, offering insights for green packaging applications.^23^ Deng et al. reported that larger cubic Boron Nitride (cBN) particle sizes improve self-bonding in pure Polycrystalline cBN (PcBN) by promoting fragmentation, plastic deformation, and strain hardening, thereby enhancing wear resistance and compressive strength.^24^ Yang et al. developed a biomass nanofibrillated cellulose composite via mechanical thermal rubber milling and hot pressing, optimizing strength through Box-Behnken design, achieving maximum MOR (31.98 MPa), MOE (3755.48 MPa), and IB (0.98 MPa) under ideal conditions of 5.6 h of grinding, 202 °C pressing, and 21 min pressing time.^25^

Zhang et al. highlighted that binderless fiberboard research faces industrial scalability challenges due to limited understanding of self-bonding mechanisms, emphasizing the need to explore lignin and furan roles during hot pressing, as well as advanced methods like laccase-mediator systems and pretreatment techniques.^26^ Wang et al. elucidated the self-bonding mechanism of bamboo binderless boards, showing that molding press temperature and time influence lignin linkages, S/G ratios, and demethoxylation, achieving optimal IB strength (0.98 MPa) at 180 °C for 20 min due to lignin’s thermal softening.^27^ Recently, Zhang et al. successfully prepared biobased lignocellulose self-bonding composites with superior bond strength, hardness, and water tolerance using hydroxyl-to-aldehyde interactions and water plasticization, making them competitive with adhesive-based materials across various tree species.^28^

This study focuses on enhancing the mechanical properties of BNC through a facile hydrophobic-barrier-patterned hot-press drying technique without the presence of binder like high content of lignin, cross-linker, and hemicellulose, Figure S1. Table S1 discloses the sample nomenclature and drying method parameters and setting. By leveraging the self-bonding mechanism in a 4-fold BNC structure, this approach investigates the effects of carbon fiber and Teflon sheet hydrophobic barriers on tensile strength, morphology, and surface characteristics via a self-bonding mechanism as a facile start to prepare biomaterial before introduction into layer-by-layer nanocomposite fabrications or in any advanced composite strategies. The goal is to address the limitations of conventional BNC drying processing methods, enabling the development of mechanically robust and sustainable materials tailored for applications in structural nanocomposites, flexible electronics, and biocompatible scaffolds.

Here, as shown in Figure 1, adapted patterns were conceived when using hydrophobic barriers and the mechanical property results when testing its tensile strength and strain in triplicate per sample. Using scanning electron microscopy (SEM), in Figure 1a, the BNC sample dried using OD showed no obvious pattern, while in Figure 1b, it adapted a square with slightly deep sides pattern from the carbon fiber orientation and, in Figure 1c, it also adapted the Teflon sheet surface showing repeating small square patterns. In Figure 1d, the mechanical properties of BNC were notably enhanced using the hydrophobic-barrier-patterned hot-press drying technique; here, the tensile test revealed a significant improvement in the tensile strength of BNC samples pressed with carbon fiber at 120 °C (HP/CF_120), achieving 43.91 N/mm^2^, a 13.84% increase compared to the control sample (OD_70), which measured 38.57 N/mm^2^ and 43.87% higher than the dried Teflon sheet barrier BNC sample (HP/Tef_100). Interestingly, samples pressed at lower temperatures with carbon fiber barrier (HP/CF_100, HP/CF, 110) and with higher temperatures with Teflon sheet barrier (HP/Tef_110, HP/Tef_120) exhibited significantly lower tensile strengths, opening further optimization for future studies.

**Figure 1:**
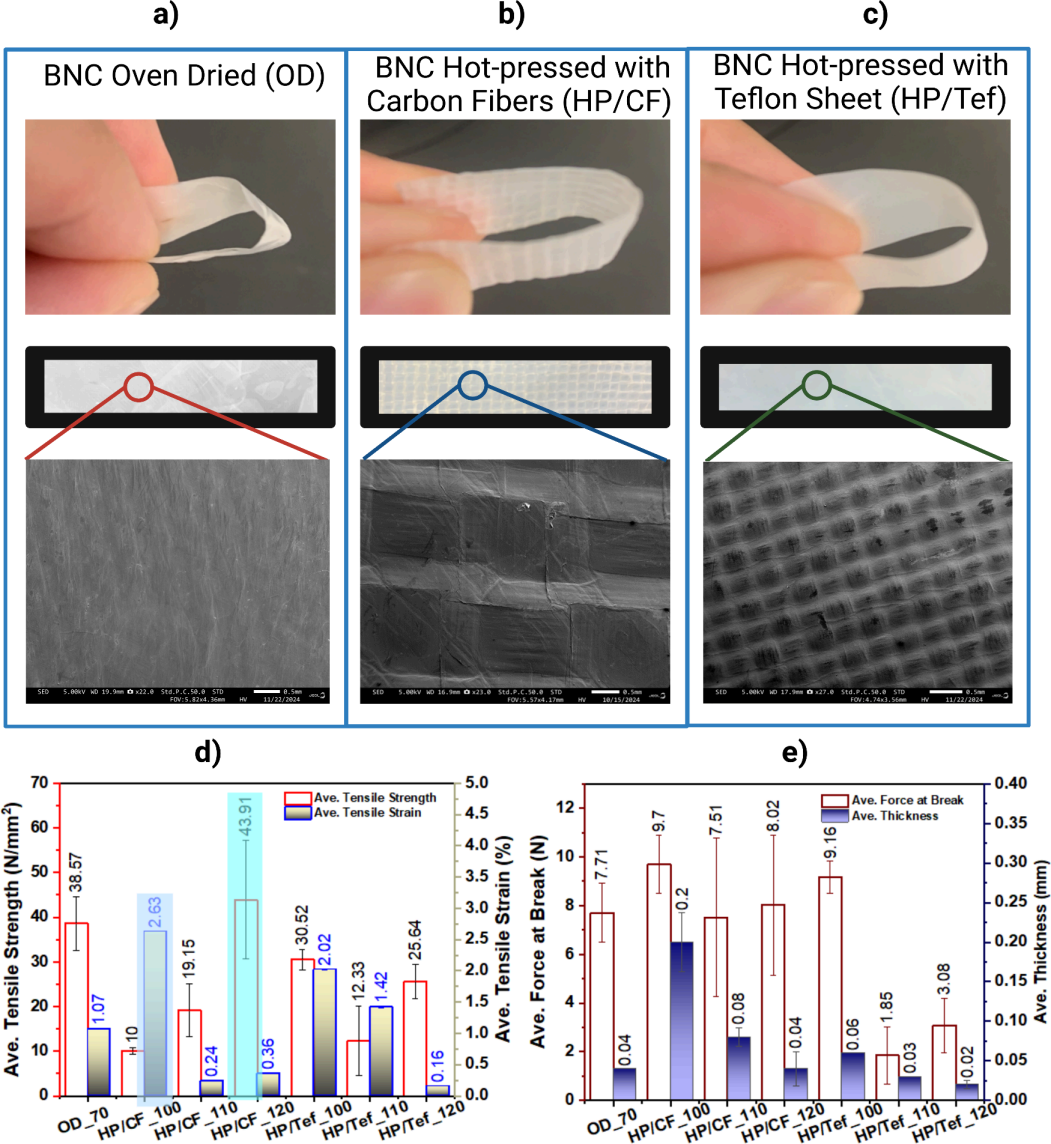
(a, b, c) Physical assessment using visual inspection and SEM at 0.5 mm scale and (d, e) mechanical property testing of the adapted patterned from hydrophobic barriers of bacterial nanocellulose.

Thus, this study focuses on the highest tensile strength results among HP/CF and HP/Tef samples and other related properties affected by the self-bonding mechanism. HP/CF_120 tensile strength enhancement can be attributed to the self-bonding mechanism facilitated by the pressing process, where tight interlocking along with friction forces and hydrogen bonding between nanocellulose fibers were responsible for the improved mechanical properties.^29^ However, unlike the carbon fiber effects for HP/CF_120, tensile strain at 0.36% and OD_70 at 1.07%, it seems that the Teflon sheet hydrophobic barrier induces a high elastic modulus as observed with HP/Tef_100 at 2.02%, which indicates that the linear slope of the stress–strain is indicative of the stiffness of the material, and the steeper the slope, the higher is the modulus of elasticity and the stiffer is the material.^30^ These results corroborate earlier studies demonstrating that pressing enhances the mechanical integrity of nanocellulose materials by improving fiber alignment and densification.^25–27^ Furthermore, the thickness and force at break of the samples played a critical role in determining mechanical properties, as shown in Figure 1e. Both HP/CF_120 and OD_70 had average thicknesses of 0.04 mm, yet the tensile strength of HP/CF_120 was markedly higher due to the influence of the carbon fiber barrier in enhancing the self-bonding mechanism and fiber packing even with exposure to higher force at break at 8.02 N against OD_70 force at break at 7.71 N. Table S2 discloses the stiffness of the samples, and Figure S2 shows that the stress–strain curve, which highlights that material choice for hydrophobic barriers, significantly influences the mechanical properties of the final BNC product. Table S3 shows a promising result in comparison to HP/CF_120 tensile strength and % strain to other existing known thermoplastic polymers.

The results show that using a hot press with a hydrophobic barrier carbon fiber is better for heat transfer and even pressure distribution across the BNC during drying on a hot press because it has higher thermal conductivity than a Teflon sheet.^31,32^ Carbon fibers facilitate a more controlled moisture release, preventing excessive water retention or rapid evaporation, which can cause internal defects. This might make the cellulose matrix denser and better aligned, which boosts hydrogen bonding and self-bonding effects and leads to higher mechanical strength at high temperatures. In addition, this work concentrated on the four-layer folding of the BNC. The little interfacial spaces created by these stacking folds serve as venting conduits, letting water vapor evaporate gradually. The use of hydrophobic barrier sheets, such as Teflon (Tef) sheets and carbon fiber (CF), changed the direction of the vapor as it dried. The carbon fiber’s multiple interwoven threads allow for controlled vapor diffusion. Conversely, nonporous Teflon encouraged moisture to depart largely around the borders of the sample, therefore preventing too much water retention at the contact surfaces.

The morphological analyses provided additional insights into the structural modifications induced by the pressing process, as shown in Figure 2. Scanning electron microscopy (SEM) showed that the nanofiber diameter (NFD) of HP/CF_120 increased by 12.5%, averaging 49.53 ± 2.46 nm, Figure 2b, compared to OD_70 with NFD at 44.01 ± 1.46 nm, Figure 2a, and a 26.97% increase compared with HP/Tef_100 with NFD at 39.01 ± 0.96 nm, Figure 2c. This increase is indicative of packing due to the pressing technique and fiber realignment as also shown in Figure S3 where the fiber direction changes.^33,34^ The fracture surface of HP/CF_120 revealed partial layer separation, Figure 2b.1; this also confirms a weak spot of the self-bonding mechanism, yet the other layers kept it to ensure overall structural integrity, contributing to enhanced tensile strength. Conversely, samples pressed with Teflon barrier HP/Tef_100 showed a more distinctive layer separation, which negatively affected their mechanical properties, Figure 2c.1. The BNC dried sample, OD_70, Figure 2a.1, shows some compartment type fractures, yet the fibers in between proved that a self-bonding mechanism exists between fibers to fibers. These linings signify the self-bonding mechanism that distributes stress across the fiber network, preventing abrupt mechanical failure. In comparison, HP/CF_120 samples exhibited more aligned fiber orientation and less aggregation, further supporting the role of hydrophobic barriers in enhancing structural cohesion. In contrast, HP/Tef_100 samples showed reduced fiber entanglement and increased layer-by-layer separation, which undermined their mechanical performance. These findings underscore the critical interplay between fiber alignment, barrier material, and processing conditions in determining BNC’s structural properties and highlights the importance of both the barrier material and pressing conditions in promoting uniform fiber orientation and achieving mechanical stability. It also proves that the pressing temperature under the hot press condition is one of the most important manufacturing parameters affecting the properties of fibers.^11,26^

**Figure 2:**
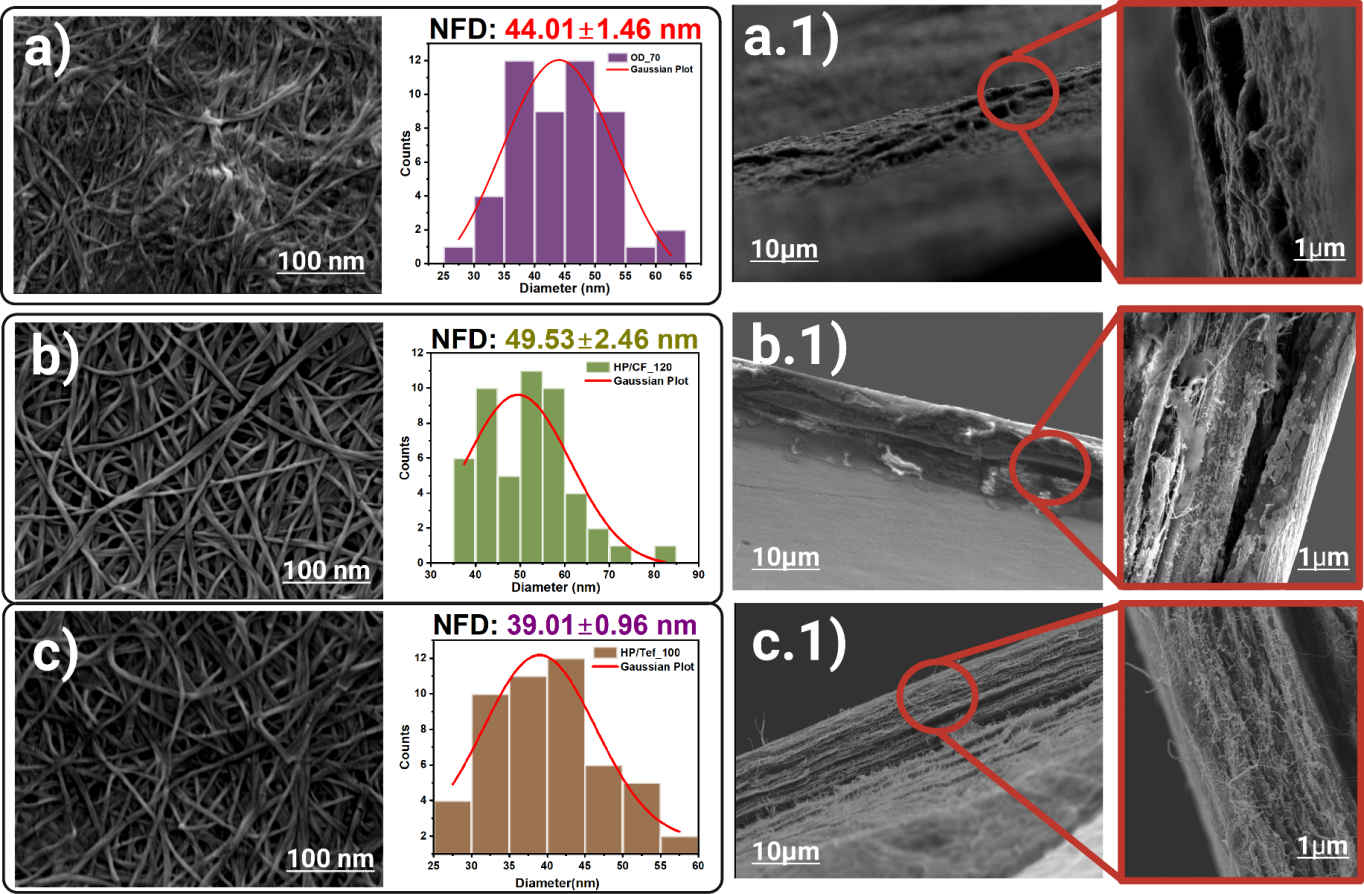
(a, b, c) Surface, before tensile test, with average nanofiber diameter (NFD) and (a.1, b.1, c.1) fracture after tensile test analysis of 4-fold bacterial nanocellulose.

The surface roughness and elevated surface characteristics of the samples were examined using KLA-Zeta and atomic force microscopy (AFM) as shown and disclosed in Figure 3 and Table S4. HP/CF_120 exhibited smoother surfaces with reduced roughness (10.3 nm) compared to that of OD_70 (12.10 nm) as disclosed by AFM images. This reduction in roughness is attributed to the alignment and compaction of the fibers facilitated by the carbon fiber barrier. Heatmaps further revealed that HP/CF_120 exhibited more elevated surface regions as conceived by the inherent pattern, Figure 3b.1, represented by intense red and yellow areas, which contribute to the material’s resistance to mechanical stress by enhancing fiber entanglement and self-bonding. However, OD_70, Figure 3a.1, shows slight intensities of red and yellow color with a strong intensity of blue color, while HP/Tef_100, Figure 3c.1, has the red and yellow colors well distributed and the intensities are almost the same as that in blue, which indicate much lower surface elevation as per the heatmap revealed. The elevated surface was confirmed by assessing the maximum peak of samples in which HP/CF_120 measured at 4.87 μm compared to OD_70 at 4.77 μm and HP/Tef_100 at 4.71 μm as disclosed in Table S4. Elevated surfaces are critical for maintaining structural integrity under load, as they distribute stress more evenly across the material delaying localized failure^35^ and the heatmap is a straightforward technique to assess it.^36,37^

**Figure 3:**
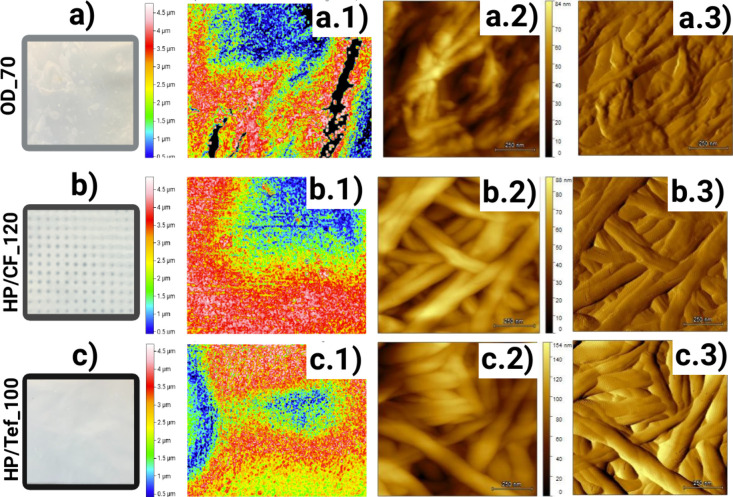
Digital images of dried BNC samples using oven drying at 70 °C (a), hotpress with carbon fiber hydrophobic barrier at 120 °C (b), and hotpress with Teflon sheet hydrophobic barrier at 100 °C (c). A KLA-Zeta 20 analyzer was employed, using a heatmap, to evaluate the elevated surfaces (a.1, b.1, and c.1). AFM was used to assess the surface roughness (a.2, b.2, c.2) and fiber entanglement (a.3, b.3, c.3) at 1 μm scan size at 250 nm.

In contrast, Teflon sheet barriers increased surface roughness with HP/Tef_100 samples recording a roughness value of 19.40 nm. The higher roughness can be linked to the inherent pattern of the Teflon barrier, which possibly hinders effective fiber alignment and bonding. In Table S4, under AFM data, HP/Tef_120 exhibited the deepest pits (79.20 nm), suggesting greater fiber gaps and weaker structural integrity. In comparison, HP/CF_120 had a maximum pit depth of 55.20 nm and OD_70 had one of 38 nm, which correlated with its improved mechanical performance and uniform surface morphology. The AFM images also showed the estimated maximum length at 250 nm scale bar of bacterial nanocellulose dried samples with OD_70 at 84 nm, Figure 3a.2, HP/CF_120 at 88 nm, Figure 3b.2, and HP/Tef_100 at 154 nm, Figure 3c.2. Furthermore, the surface AFM images show a less defined nanofibrillar network with OD_70, Figure 3a.3, compared with the clear nanofibrillar network of HP/CF_120, Figure 3b.3, and HP/Tef_100, Figure 3c.3.

The structural integrity of the BNC samples was further analyzed using Fourier transform infrared spectroscopy (FTIR) and X-ray diffraction (XRD), as shown in Figure 4. Figure 4a shows the overall FTIR spectra of dried bacterial nanocellulose, wherein the appearance of various functional groups comprised the structure of the produced nanocellulose. The absorption of bands in the region of 1200–900 cm^–1^ are the most intensive, and thus, the samples under study can be considered as relatively pure BC. The characteristic IR absorption bands for BNC are found at 896 cm^–1^ (C–O–C stretching of β-(1–4)-glycosidic linkages) indicating the amorphous components; 1160, 1108, 1056, and 1031 cm^–1^ (C_1_–O_4_H, C_2_–O_2_H, C_3_–O_3_H, and C_6_H_2_–O_6_H vibrations, respectively); 1370 cm^–1^ (CH_2_ bending) indicating the crystallinity; 1428 cm^–1^ (symmetric CH_2_ bending vibrations) indicating the crystallinity and 3340 cm^–1^ (intramolecular hydrogen bond for (O_3_–H–O_5_)).^38–41^ Two absorption bands at 1652 cm^–1^ and 1541 cm^–1^ (amide I and amide II, respectively) are the characteristic bands of proteins in biosample spectra.^42^ In the spectra of BC samples washed with water, band at 2894 cm^–1^ also indicated some impurities.^43^

**Figure 4:**
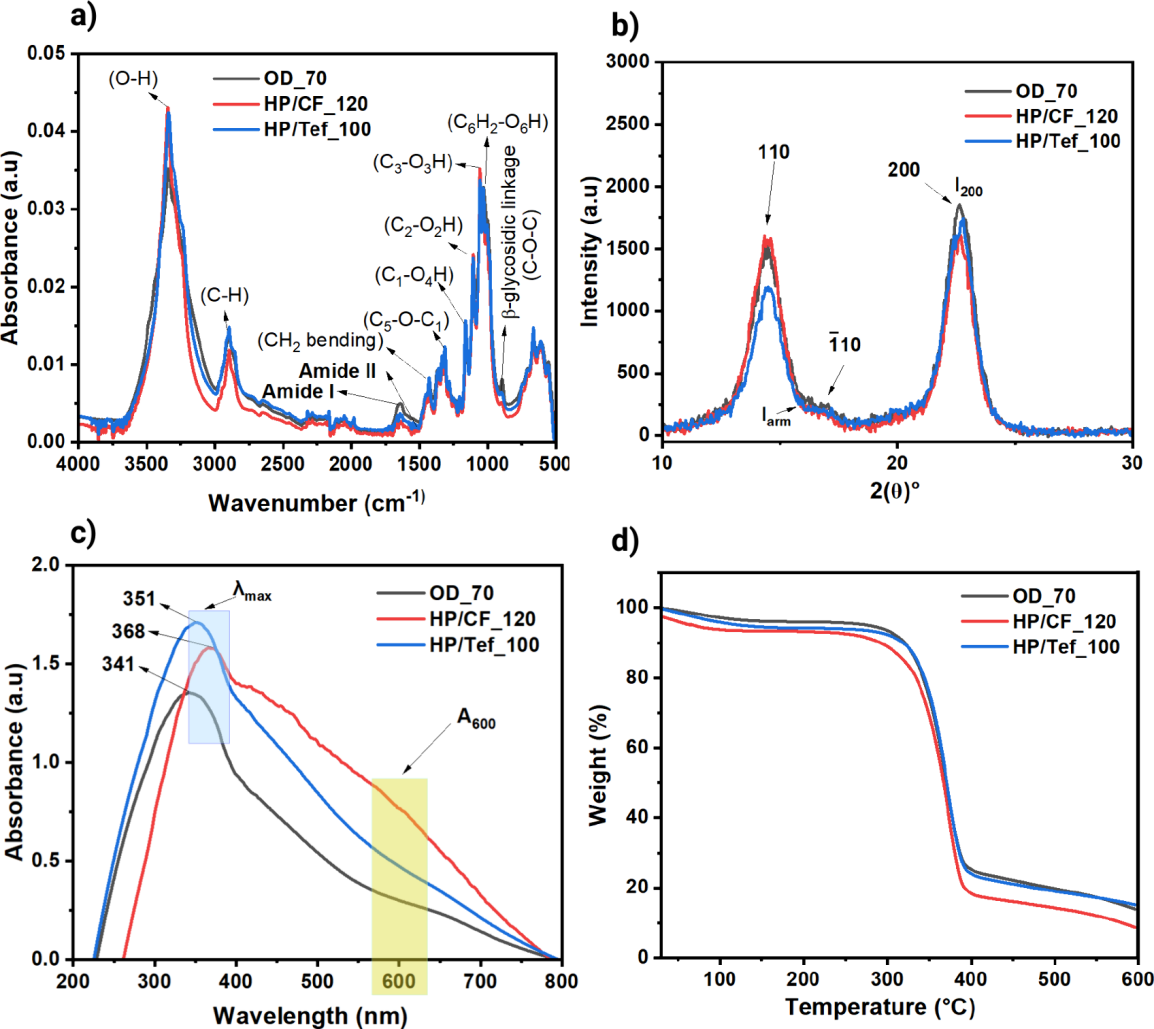
FTIR spectra (a), XRD (b), UV–vis (c), and TGA (d) thermograph of dried bacterial nanocellulose samples.

In Table S5, HP/CF_120 exhibited the highest purity of 95.41%, despite lower dry weight, 1.23 g/L, recovery from the never-dried phase when compared to OD_70 at 94.45% purity with 1.27 g/L dry weight and HP/Tef_100 at 94.20% with 1.56 g/L dry weight. A clear relationship between the interaction of hydroxyl groups and crystallinity in cellulose has been established by using FTIR.^44–46^ Here, we found that when processing with carbon fiber HP/CF_120 increases the empirical crystallinity index at 2.40 compared with HP/Tef_100 at 1.85 and OD_70 at 1.31. The crystallinity band was observed for OD_70, HP/CF_120, and HP/Tef_100 at 1427 cm^–1^, while the amorphous band was observed at 896 cm^–1^ for OD_70 and shifted to 900 cm^–1^ for HP/CF_120 and HP/CF_Tef_100, Figure S3.^44^ It has been found that crystallinity decreases as the intensity of the absorption at crystallinity band decreases, whereas the intensity of the absorption at amorphous band increases.^39,47,48^ Hydrogen bond intensity (HBI) can also be used to interpret qualitative changes in cellulose crystallinity; for example, crystallinity decreases with the increase of HBI. An increase in HBI represents an increase in hydrogen bonding between certain hydroxyl functions in the cellulose, which is typical of the conversion of cellulose I to cellulose II; even though this represents a decrease in the overall crystallinity index.^49^ In our study, we measured the HBI by using the ratio of the integrated area at 3340 cm^–1^ for OD_70 and HP/Tef_100 and 3345 cm^–1^ for HP/CF_120 (Figure S4) and 1334 cm^–1^ for OD_70 and HP/CF_120 and 1336 cm^–1^ for HP/Tef_100 (Figure S5). Both hot press samples (HP/CF_120 and HP/Tef_100) show reduced HBI, 28.88 and 26.38, respectively, compared with OD_70 at 34.06, indicating that using hydrophobic barriers disrupts hydrogen bonding, which clearly contributed to changes in crystallinity, inducing a greater effect on mechanical properties via the self-bonding mechanism. Meaning, the increase of LOI with a decrease in HBI indicated a significant increase in the crystalline fraction in cellulose. This was attributed to preferential digestion of the more disordered cellulose regions prior to engaging yhe highly crystalline substrate.^50^

Figure 4b shows the X-ray diffraction patterns of BNC samples confirmed the presence of three distinct peaks at 2θ values of 14.5°, 16.7°, and 22.7°, consistent with previous research,^51–53^ which has the following crystallographic planes of 110, 110, and 200, respectively. Representing typical diffraction peaks of type I cellulose and reflecting the crystalline and amorphous structure of BNC components, the CrI stands as a crucial parameter for BNC and its derived compounds.^54^ According to Segal’s formula, the crystallinity of BNC produced and dried using oven-drying without a hotpress and hydrophobic barrier, OD_70, reached 89.21%, while for BNC produced and dried using a hotpress with a carbon fiber hydrophobic barrier, HP/CF_120, yielded higher at 90.38%; however, the Teflon sheet hydrophobic barrier, HP/Tef_100, seems to reduce the degree crystallinity due to disruption of the ordered crystalline regions, which reached 81.88% (see Table S6). The HP/CF_120 result is much higher and surpassed that of BNC produced in the previous studies with single layer analysis.^55–57^ These findings suggest that drying methods and barriers could influence the crystallinity of BNC.^58^ The XRD data revealed that HP/CF_120 had the largest average crystallite size, 6.81 nm, further supporting its superior mechanical performance. Larger crystallite sizes correspond to more organized and well-defined crystalline regions, which enhance load transfer and reduce strain.^44^ Conversely, HP/Tef_100 exhibited the smallest crystallite size, 5.60 nm, limiting its mechanical strength since the appearance of more inherent patterns disrupted the ordered crystalline regions more, as shown in Figure 1. This disruption can create a less uniform and more amorphous structure on the bacterial cellulose surface.^59^

Figure 4c of the BNC samples revealed an additional improved property of the carbon fiber hydrophobic-barrier-patterned pressing technique. In Table S7, HP/CF_120 exhibited a higher opacity, 17.82%, and reduced transparency compared to OD_70 at 8% and HP/Tef_100 at 7.09%. The compact structure achieved by more elevated surfaces as shown by surface roughness through carbon fiber pressing contributed to these optical characteristics. Meaning, for opaque surfaces, the light that reflects off them changes with the surface’s orientation.^60^ However, in thermal properties, Figure 4d, the higher temperature set for HP/CF_120 results in less residue and weight loss, indicating a cleaner material with fewer impurities.^61,62^ Despite this, its thermal stability remains almost the same as OD_70 and HP/Tef_100. Fewer impurities suggest a higher crystallinity that leads to improved tensile strength for HP/CF_120. All data for thermal analysis was disclosed in Table S8.

The hydrophobic barrier-patterned hot-press drying technique represents a significant step forward in tuning the properties of bacterial nanocellulose. By leveraging self-bonding mechanisms, these findings open the door to diverse applications for BNC, including structural composites, flexible electronics, and biocompatible scaffolds. Future studies should focus on optimizing pressing conditions and exploring alternative hydrophobic barriers to further expand BNC’s potential in sustainable, high-performance material solutions.

## Supplementary Material



## Data Availability

Data available on request from the authors.

## Notes

Materials and methods including figures for experimental setup, stress vs strain and load vs displacement curves, the fiber directionality; peak area (P.A) and peaks designation for assigned cellulose functional groups; peak area and peak designation for BNC impurities; characterization and testing; stiffness of the samples; comparison of mechanical properties to other existing known thermoplastic polymers; surface topography and roughness measurement; dry weight,% purity, and IR crystallinity indexes; D-spacing and % crystallinity; transparency and opacity; thermal degradation, weight loss, and residual weight (PDF)
