# Supplementary material for: Insight on the Mechanical Properties of Facile Hydrophobic-Barrier-Patterned Bacterial Nanocellulose via Self-Bonding Mechanism
Source: ACS Nanosci Au. 2025 Mar 20;5(3):128–36. doi: 10.1021/acsnanoscienceau.4c00077 (PMC12183586; doi:10.1021/acsnanoscienceau.4c00077)
Supplement: Supplementary file 1 [file ng4c00077_si_001.pdf]

SUPPORTING INFORMATION

Insight on the Mechanical Properties of Facile Hydrophobic-Barrier-Patterned Bacterial Nanocellulose via Self-Bonding Mechanism

δ [1]Maurelio Cabo Jr., δ [2]Nitin More , [2]Jeffrey R. Alston, [1]Eric Laws, [2]Rutujaa Kulkarni, **[2]Ram V. Mohan, *[1]Dennis R. LaJeunesse

[1]Department of Nanoscience, Joint School of Nanoscience and Nanoengineering, University of North Carolina Greensboro, Greensboro, North Carolina, 27402, USA

[2]Department of Nanoengineering, North Carolina Agricultural and Technical State University, Greensboro, North Carolina, 27401, USA

Corresponding Authors: *drlajeun@uncg.edu, **rvmohan@ncat.edu

δ M.C.Jr., and δ N.M contributed equally to this work

A. Materials and Methods

1. Materials.

(D+)Glucose, Yeast extract, NaOH pellets, Na_2HPO_4, Agar powder, and Peptone were purchased from Fisher Scientific (Thermo Fisher Scientific, Waltham, MA, USA). Pellicles of BC were collected from cultures of Gluconacetobacter hansenii (ATCC 23769, American Type Culture Collection, Manassas, VA, USA). Carbon fiber was purchased from Toray Industries, Inc., USA, which is plain weave (T300B-3000-40B), with thickness of 0.24 mm and PTFE Teflon Sheet (12”x16”) was purchased from Ubrand, USA.

2. Fabrication of Bacterial Nanocellulose.

Figure S1(a) shows the fabrication of BNC in HS media in static culture, pretreatment of BNC in concentrated NaOH, and drying techniques. Here, we culture the Gluconacetobacter hansenii bacteria in Hestrin-Schramm (HS) media with the following composition: 2% (w/v) D-glucose, 0.5% (w/v) yeast extract, 0.5% (w/v) peptone, 0.27% (w/v) Na_2HPO_4, and 0.125% (w/v) citric acid **[1-2].** To inoculate the bacteria strain cells, prepare a separate agar plate in a petri dish (85 mm x 15 mm) with the same composition of HS media and 2% agar, and incubate it for 2 days at a temperature of 30 °C. Next, we transfer a pea-sized colony of cell from the inoculation petri dish and culture it in 500 mL of media in an anchor hocking glass 9” by 13” baking dish in the incubator with controlled temperature of 25 °C. After 10 days, we processed the pellicles in a 0.1M concentration of NaOH at a set temperature 95°C for 1 hour to remove all bacteria and bacterial biofilm materials other than the BC matrix. We then wash the pellicles with distilled water until we achieve a neutral pH (7.0). We store the pellicles in DI water at room temperature.

3**.** BNC Drying Technique using Hotpress with selected hydrophobic barriers.

Using Hotpress Model 25-12H, USA, at 35 psi, the wet bacterial nanocellulose with dimension 9 x 13 inches was folded into four and sandwich between the 7x7 cm size cut hydrophobic barriers (Carbon

fiber/Teflon). Table S1 shows the nomenclature, settings and parameters used per each experiment. Fig. **S**1 (a,b) shows the experimental method used for this study. The hot press has a digitally set temperature. It was also the actual temperature during its entire run per sample as shown also digitally.

<table>
<tr><td colspan="8">Table S1: Samples Nomenclature and Drying Method Parameters and Settings</td></tr>
<tr><td rowspan="2">Samples</td><td rowspan="2">Nomenclature</td><td rowspan="2">Drying Technique</td><td rowspan="2">Hydrophobic Barrier</td><td colspan="2">Temperature Setting (°C)</td><td colspan="2">Drying Time</td></tr>
<tr><td>*Set</td><td>*Actual</td><td>During (h)</td><td>After (h)</td></tr>
<tr><td>1</td><td>OD_70</td><td>Oven Drying (OD)</td><td>-</td><td>70</td><td>70</td><td>5</td><td>0.5</td></tr>
<tr><td>2</td><td>HP/CF_100</td><td>Hot-press (HP)</td><td>Carbon Fiber (CF)</td><td>100</td><td>100</td><td rowspan="6">0.5</td><td rowspan="6">0.5</td></tr>
<tr><td>3</td><td>HP/CF_110</td><td>Hot-press (HP)</td><td>Carbon Fiber (CF)</td><td>110</td><td>110</td></tr>
<tr><td>4</td><td>HP/CF_120</td><td>Hot-Press (HP)</td><td>Carbon Fiber (CF)</td><td>120</td><td>120</td></tr>
<tr><td>5</td><td>HP/Tef_100</td><td>Hot-Press (HP)</td><td>Teflon Sheet (Tef)</td><td>100</td><td>100</td></tr>
<tr><td>6</td><td>HP/Tef_100</td><td>Hot-Press (HP)</td><td>Teflon Sheet (Tef)</td><td>110</td><td>110</td></tr>
<tr><td>7</td><td>HP/Tef_100</td><td>Hot-Press (HP)</td><td>Teflon Sheet (Tef)</td><td>120</td><td>120</td></tr>
</table>

*Set and Actual temperature in hot press originally in Fahrenheit but was converted into Celsius degree in the table above.

The BNC production yield was computed by the following equation:

$$BNC\ Production\ yield = \frac{dried\ BNC\ (g)}{Culture\ Medium\ Volume\ (L)} \quad (1)$$

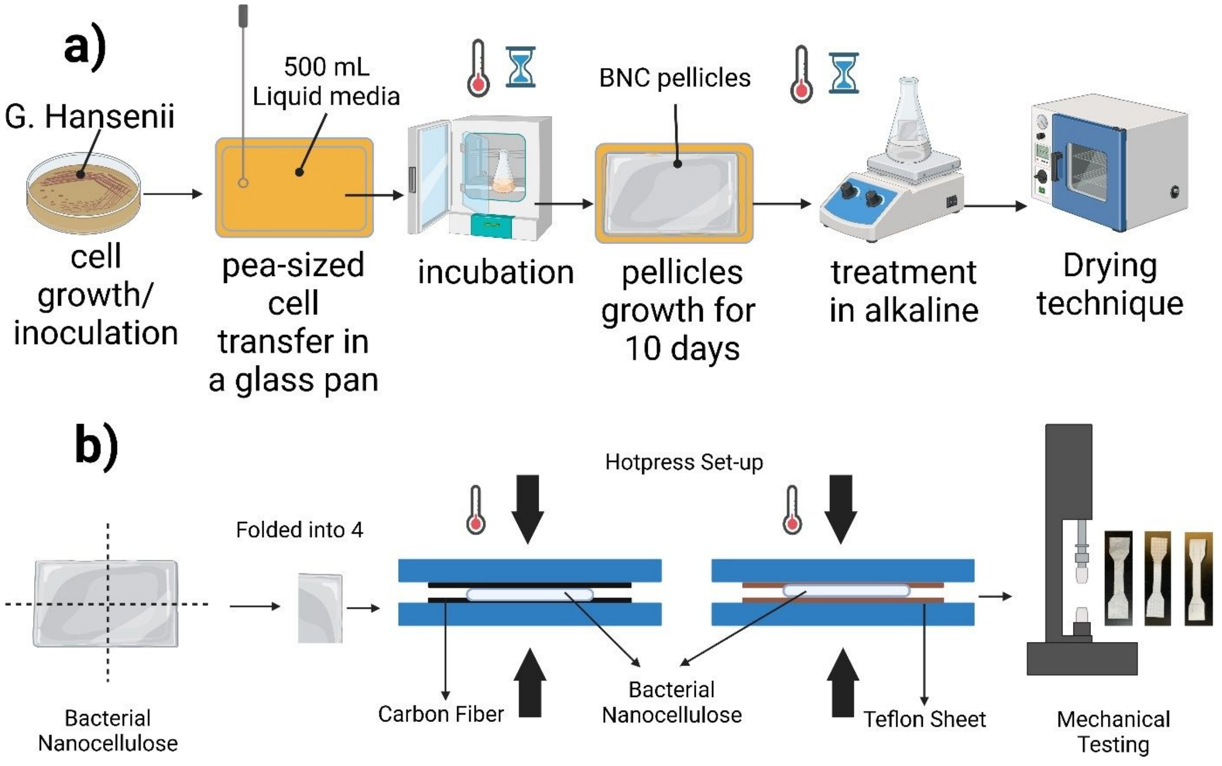

Figure S1. Growing Bacterial Nanocellulose (a) and using Hot-press with hydrophobic barriers for drying technique (b).

B. Characterization and Testing

1. Mechanical Property Test. The oven -dried pellicles underwent tensile strength testing using a universal testing machine (Torbal -Poland). Due to the brittleness, dried samples were manually cut based on the mold from Dogbone stamp ISO 37 **[3]**. The thickness of each strip was measured using a digital caliper. Subsequently, the samples were secured between two clamps with a gauge length of 50 mm and subjected to a force at 10 mm/min rate under ambient temperature (23 °C) until the sample broke. Maximum stress and elongation at break were determined using the data from the Force Manager software. Stress (MPa) was calculated as the ratio of loading force (expressed in Newtons, N) to the cross -sectional area of the sample (A). Strain (%) was calculated as $\Delta L/L_o \times 100\%$, where L_o represents the initial length and ΔL signifies the extension from the starting point or the recorded maximum displacement. The mechanical tests represent the average of three experiments ± standard deviation (SD).

2. Morphological Analysis. The scanning electron micrographs were obtained using JEOL JSM-IT800 Schottky FESEM (Zeiss, Jena, Germany). Nanofibers diameters were measured using ImageJ software (U.S. National Institutes of Health, Bethesda, Maryland, USA), 50 times per sample. Surface and fracture morphology were assessed. Fiber directionality was measured using Fiji ImageJ software wherein it computes a histogram indicating the amount of structure in each direction. The parameter setting at 0° to 180° **[4]**.

3. Elevated Surface via Heat Map Analysis. To do heat map simulation on samples' elevated surface, optical profiler was employed. This benchtop optical profiler is a non-contact, 3D microscope and surface topography measurement system, model: Zeta-20 (KLA Corporation, USA).

4. Surface Roughness Analysis. Topographical features like surface roughness and pit depth of dried bacterial nanocellulose surfaces were recorded on Asylum-3D Origin + AFM in non-contact mode using Tap-150G AFM probes purchased from Ted Pella (resonant frequencies of 150 kHZ (+/- 75 kHz), a length of 125 μm (+/- 25 μm), a width of 25 μm (+/- 5 μm) and a force constant ranging from 1.5-15 N/m). Measurements were performed at ambient temperatures, with images processed using Gwyddion software packages.

5. Percent Purity Analysis. To quantify BNC purity, IR spectra were recorded on a Fourier-transform infrared spectroscopy (FTIR) spectrometer (Agilent 670 FTIR Spectrometer, Santa Clara, CA, USA) under dry air at ambient temperature. The percentage of transmittance spectra was recorded from 4000 to 400 cm^{-1} with 64 scans in each case at a resolution of 4 cm^{-1}. For purity proportional percentage calculation of alkaline treated bacterial nanocellulose, the following equation was used based on the Yamamoto et al mass fractions equation [**5**]:

$$\%Purity = \frac{A_{Cellulose} Peaks}{A_{CellulosePeaks} + A_{ImpuritiesPeaks}} \text{ x } 100 \qquad (2)$$

The peak designations for Area of cellulose peaks ($A_{\text{cellulose peaks}}$) and Area of impurities peaks ($A_{\text{impurities peaks}}$) were based on the previous study conducted by Grube et al wherein for the first time FT-IR spectra of different origin BNC samples were recorded and analyzed to select the best method for screening and/or evaluation of the BNC quality [**6**].

The crystallinity of cellulose was measured using Empirical ''crystallinity index'' or Lateral order index (LOI) where absorption band located at 1427 cm^{-1} and absorption band located at 896 or 900 cm^{-1} were used **[7]** using the below formula:

$$LOI = \frac{A_{1427}}{A_{896 \ or \ 900}} \qquad (3)$$

Hydrogen bond intensity (HBI) can also be used to interpret qualitative changes in cellulose crystallinity, where absorption band located at 3340 or 3345 cm^{-1} and absorption band located at 1334 or1336 cm^{-1} were used **[8]** using the below formula:

$$HBI = \frac{A_{3340 \ or \ 3345}}{A_{1334 \ or \ 1336}} \qquad (4)$$

6. Percent Crystallinity Analysis. Crystallinity analysis was conducted using Rigaku SmartLab equipment with Cu-Kα radiation. A copper source was used at 40 V and 40 mA. The samples were mounted on the sample holder, and the patterns were recorded by running the instrument at a speed of 5°/ min and a 2θ range of 5°–40°. The Scherrer's formula was used to measure the crystallite size:

$$CrystalliteSize = \frac{k\lambda}{W} cos\theta \qquad (5)$$

(with a shape factor k = 0.94 was employed to determine the crystallite sizes of samples with full width at half maximum (fwhms, W) and peak centers obtained by fitting the (110) and (200) peaks at the Gaussian

function using OriginPro software; here λ is the wavelength of X-ray radiation (0.154 nm) [**9**]. The lattice spacing (d-spacing) was calculated using Bragg's equation [**10**]:

$$\lambda = 2d_{hkl} * sin\theta \quad (6)$$

The crystalline index (CrI^{XRD}) was calculated with the help of the following formula:

$$CrI = \left(\frac{I_{200} - I_{am}}{I_{200}}\right) x100 \quad (7)$$

Where I_{200} is the maximum intensity of the (200) lattice diffraction and I_{am} is the intensity diffraction at 14° (2θ) [**11**].

7. Optical Property. The dried samples were subjected to optical property analysis employing a UV-Vis Thermo Scientific NANODROP 2000C. The samples were analysed in a 10 x 45 mm, 3.5mL quartz cuvette.

8. Thermal Stability. The thermal stabilities of DES, DES/TA, and DES/TA/BNC solvent systems were characterised using a thermogravimetric analyser (TGA; Perkin Elmer STA 6000, England) within a temperature range of 30 to 600 °C at a rate of 20 °C/min under an air atmosphere.

9. Statistical analysis. Mechanical property experiment was performed in triplicate, and each response's mean value ± SD was reported. Histogram graphs, normal density distribution, FTIR, XRD, and TGA graphs were generated using OriginPro 2024b Academic Software.

**Table S2: Stiffness of the samples**	
Samples	*Young's Modulus (MPa)
OD_70	29.5 ± 0.32
HP/CF_100	3.98 ± 0.04
HP/CF_110	80.73 ± 0.16
HP/CF_120	146.92 ± 0.30
HP/Tef_100	19.53 ± 0.53
HP/Tef_110	33.4 ± 1.30
HP/Tef_120	186.16 ± 0.38

*Standard Deviation reported in RSD format

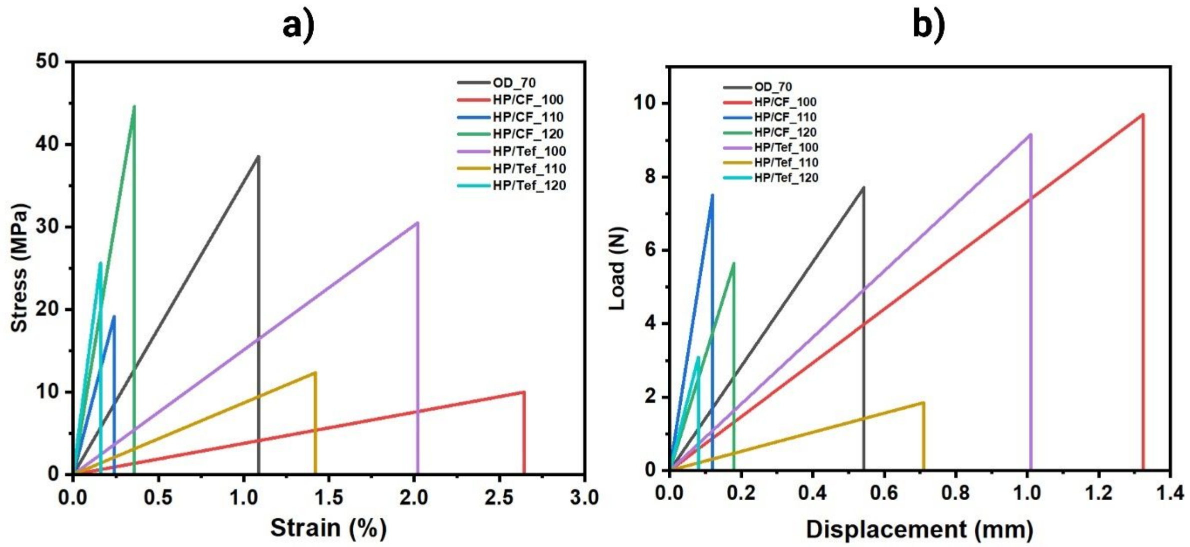

Figure S2: Stress vs Strain and Load vs Displacement curves.

**Table S3. Comparison of Mechanical Properties to other existing known thermoplastic polymers**			
Sample	Tensile Strength (MPa or N/mm^2)	Strain (%)	Reference(s)
Bacterial Nanocellulose (4-fold)	43.91	0.36	This Study
Polypropylene	26-41.4	15-700	**[12-13]**
High density polyethylene	14.5-38	2-130	**[14]**
Low density polyethylene	8.48-26.2	11-640	**[15]**
Polystyrene	34-55	1-35	**[16]**
Nylon 6	28-80	4-25	**[17]**

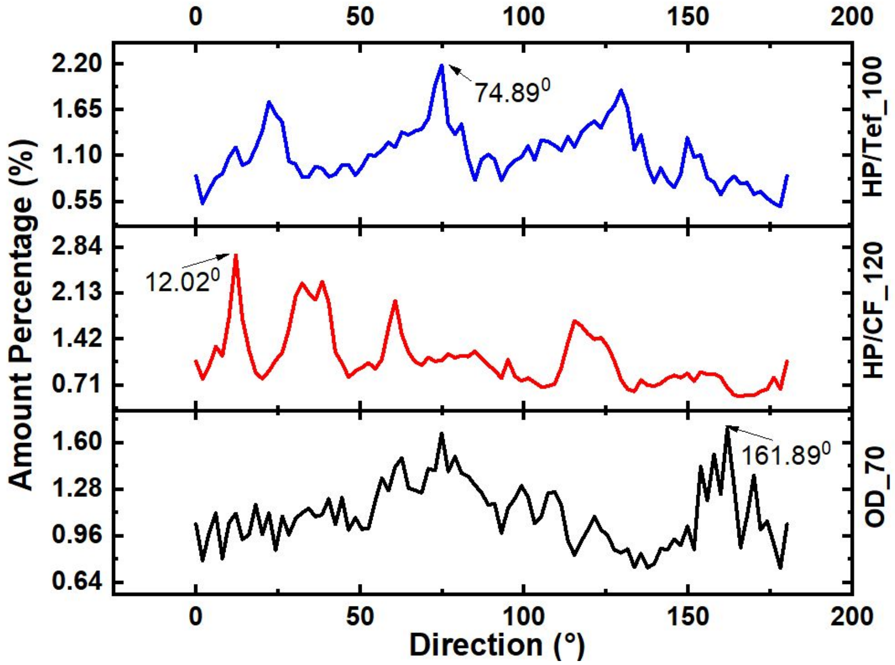

Figure S3: The fiber directionality was measured using the Fiji ImageJ software. This demonstrates how the addition of hydrophobic barriers alters the fiber directions. The OD_70 fiber direction is significantly higher at 161.89°, the HP/CF_120 fiber direction is now more concentrated at 12.02°, and the HP/Tef_100 fiber direction is at 74.89°.

Table S4. Surface Topography and Roughness Measurement

Samples	KLA Zeta Analyzer			Atomic Force Microscopy (AFM)	
	Arithmetic Mean Height (µm)	Valley Depth (µm)	Max Peak to Valley Height (µm)	Mean roughness (Sa) (nm)	Max. Pit Depth (nm)
OD_70	1.19	2.19	4.77	12.1	38.0
HP/CF_120	1.09	2.69	4.87	10.3	55.2
HP/Tef_100	0.80	2.62	4.71	19.4	79.2

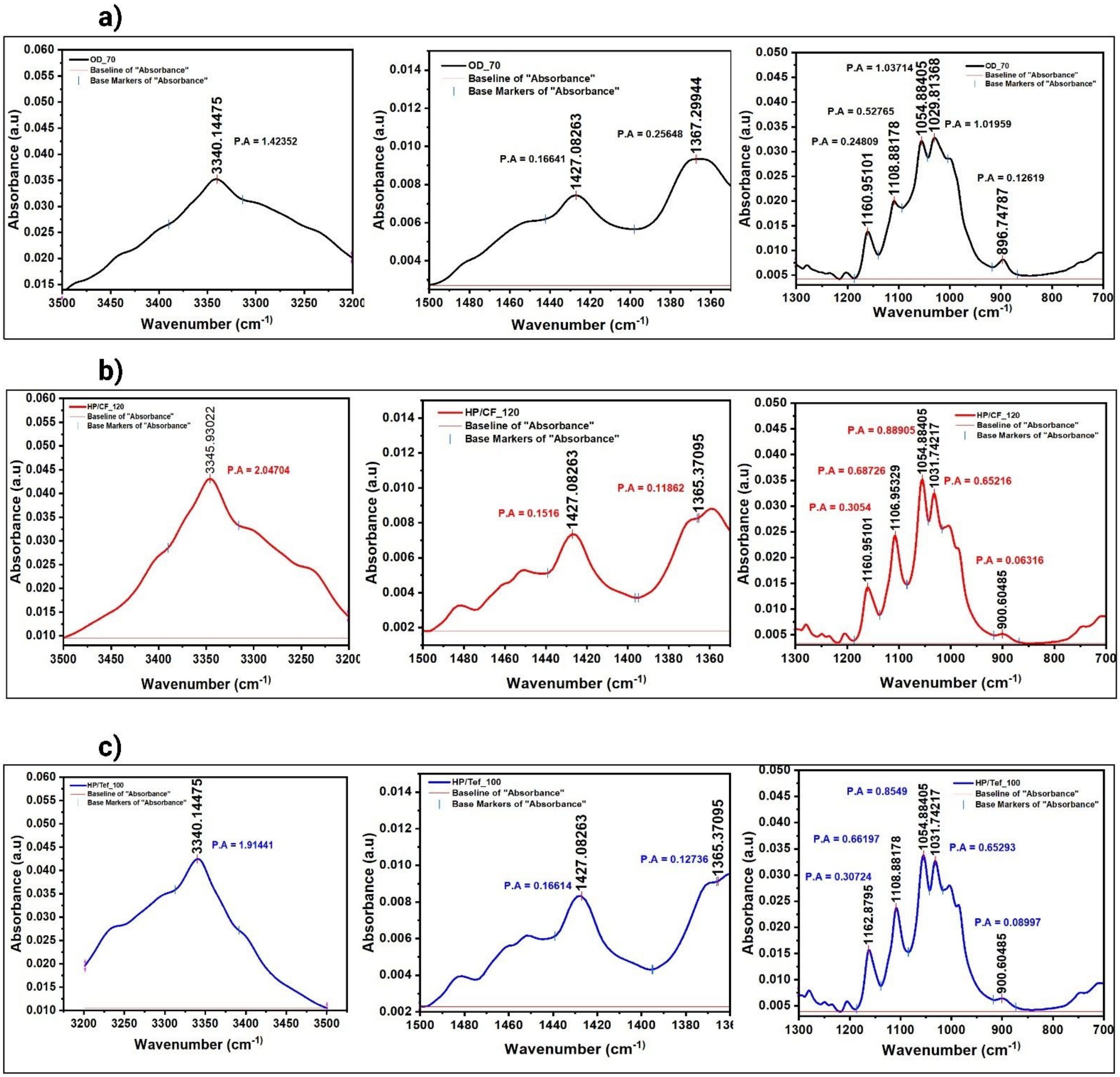

Figure S4: Peak Area (P.A) and peaks designation for assigned Cellulose functional groups of BNC for OD_70 (a); HP/CF_120 (b); and HP/Tef_100 (c). Peak designation at 3340 or 3345 cm^{-1} was used for Hydrogen Bond Intensity while peaks absorption at 1427 cm^{-1} and at 896 or 900 cm^{-1} were also used for crystallinity index or Lateral order index (LOI).

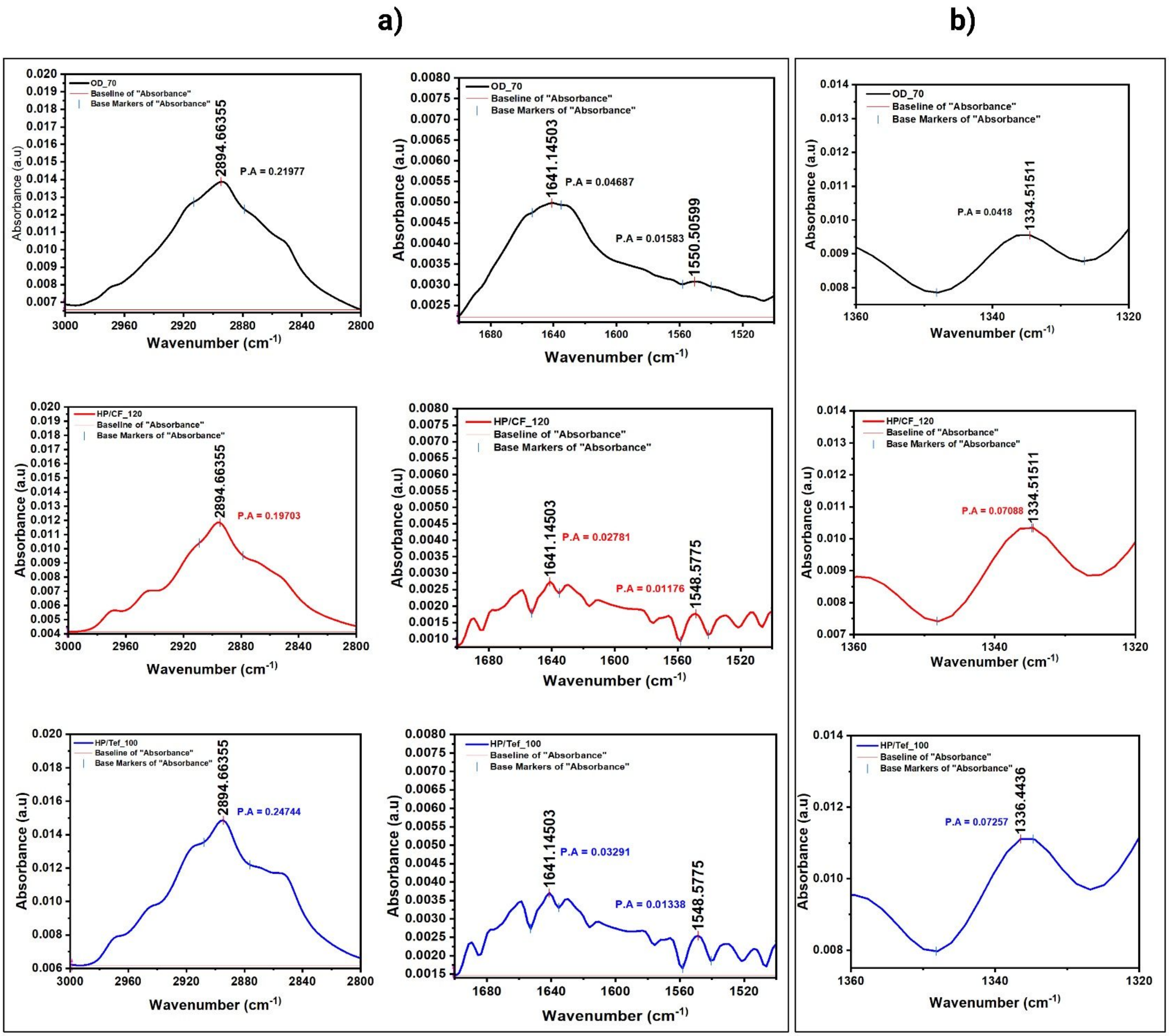

Figure S5: Peak Area (P.A) and peaks designation for BNC impurities (a) and Hydrogen Bond Intensity (b) peak designation at 1334 or 1336 cm^{-1}

**Table S5. Dry weight, % Purity and IR crystallinity indexes**				
Samples	Dry Wt. (g/L)	% Purity (IR)	Empirical Crystallinity Index (A_{1428}/A_{896})	Hydrogen Bond Intensity (HBI) (A_{3340}/A_{1336})
OD_70	1.27	94.45	1.31	34.06
HP/CF_120	1.23	95.41	2.40	28.88
HP/Tef_100	1.56	94.20	1.85	26.38

**Table S6. D-spacing and % crystallinity**					
Samples	d1	d2	d3	Ave. Crystallite Size	% Crystallinity
OD_70	0.031	0.026	0.020	6.46	89.21
HP/CF_120	0.031	0.027	0.020	6.81	90.38
HP/Tef_100	0.031	0.027	0.020	5.60	81.88

**Table S7. Transparency and Opacity**			
Samples	A_{600} (nm)	$\%T_{600}$	Opacity (%)
OD_70	0.312	48.75	8.00
HP/CF_120	0.873	13.40	17.82
HP/Tef_100	0.468	34.04	7.09

Table S8: Thermal degradation, weight loss and residual weight

	[a]T_o (°C)	[b]$T_{initial}$ (°C)	[c]T_p (°C)	[d]T_{end} (°C)	[e]W_{L-1} (%)	[f]W_{L-2} (%)	[g]RY (%)
OD_70	125.91	333.32	371.45	393.66	70.07	10.95	13.91
HP/CF_120	91.86	332.19	373.41	391.25	73.68	9.46	8.67
HP/Tef_100	125.52	339.56	372.11	394.28	69.77	8.35	15.14

a – onset Temperature

b – Temperature for initial degradation

c – peak Temperature measured from Derivative weight

d – Temperature for endset degradation

e – weight loss for 1st degradation

f – weight loss for 2nd degradation

g – residual yield

References:

1. Gorgieva, S., Trcek, J. Bacterial Cellulose: Production, Modification and Perspective in Biomedical Applicantions. Nanomaterials Basel, **2019**, 9, 1352.
2. Warren, R., Lajeunesse, D., "Characterization of Hydrothermal Deposition of Copper Oxide Nanoleaves on Never-Dried Bacterial Cellulose," *Polymers,* **2019,** 11, 1762.
3. ISO 37: Rubber, vulcanized or thermoplastic — Determination of tensile stress-strain properties. Link: https://www.mecmesin.com/standard/iso-37. Accessed: November 24, 2024.
4. Directionality. ImageJ. Link: https://imagej.net/imagej-wiki-static/Directionality. Accessed: March 10, 2025.
5. Yamamoto, H., Horii, F., Hirai, A. "In situ crystallization of bacterial cellulose II. Influences of different polymeric additives on the formation of celluloses Iα and Iβ at the early stage of incubation," *Cellulose*, **1996,** 3, 229-242.
6. Grube, M., Shvirksts, K., Denina, I., Ruklisa, M., Semjonovs, P. "Fourier-transform infrared spectroscopic analyses of cellulose from different bacterial cultivations using microspectroscopy and a high-throughput screening device," *Vibrational Spectroscopy*, **2016,** 84, 53-57.
7. Salem, K. S., Kasera, N. K., Rahman, M. A., Jameel, H., Habibi, Y., Eichhorn, S. J., French, A. D., Pal, L., & Lucia, L. A. Comparison and assessment of methods for cellulose crystallinity determination. *Chemical Society Reviews*, **2023,** *52*, 6417–6446.
8. Xiao, L., Lin, Z., Peng, W., Yuan, T., Xu, F., Li, N., Tao, Q., Xiang, H., & Sun, R. Unraveling the structural characteristics of lignin in hydrothermal pretreated fibers and

manufactured binderless boards from Eucalyptus grandis. *Sustainable Chemical Processes*, **2014,** *2*, 9.

9. A. Patterson. “The Scherrer formula for X-ray particle size determination,” *Phy. Rev.* **1939,** 10, 978.
10. Xiaohui Ju, Mark Bowden, Elvie E. Brown, Xiao Zhang, “An improved X-ray diffraction method for cellulose crystallinity measurement,” *Carbohydrate Polymers*, **2015**, 476-481.
11. Segal, L., Creely, J.J., Martin, A.E., Conrad, C.M. “An Empirical Method for Estimating the Degree of Crystallinity of Native Cellulose Using the X-Ray Diffractometer,” *Textile Research Journal*, **1959,** 29, 786-794.
12. Ku, H., Wang, H., Pattarachaiyakoop, N., & Trada, M. A review on the tensile properties of natural fiber reinforced polymer composites. *Composites Part B Engineering*, **2011,** *42*, 856–873.
13. Tensile Strength of Polypropylene. Link: https://www.shimadzu.com/an/industries/chemicals/film/tensile-strength-of-polypropyl/index.html#:~:text=Further%2C%20the%20tensile%20strength%20(average,absorbed%20energy%20is%200.49%20J. Accessed: Nov. 24, 2024
14. Ku, H., Wang, H., Pattarachaiyakoop, N., & Trada, M. A review on the tensile properties of natural fiber reinforced polymer composites. *Composites Part B Engineering*, **2011,** *42*, 856–873.
15. Overview of materials for Low Density Polyethylene (LDPE), Blow Molding Grade. Link: https://www.matweb.com/search/datasheet_print.aspx?matguid=b34a78d271064c4f85f28a9ffaf94045. Accessed: Nov. 24, 2024
16. Overview of materials for Polystyrene, Transparent Grade. Link: https://www.matweb.com/search/DataSheet.aspx?MatGUID=873cf14a45b8416ab0d6f4e2fa24e61e Accessed: Nov. 24, 2024
17. Overview of materials for Nylon 6, Film Grade. Link: https://www.matweb.com/search/DataSheet.aspx?MatGUID=6532b02c347d446b8241390cc04a3e66 Accessed: Nov. 24, 2024
